# Progress in the utilization of antenatal and delivery care services in Bangladesh: where does the equity gap lie?

**DOI:** 10.1186/s12884-016-0970-4

**Published:** 2016-07-29

**Authors:** Mohammad Habibullah Pulok, Md Nasim-Us Sabah, Jalal Uddin, Ulrika Enemark

**Affiliations:** 1 Department of Economics, Southeast University, Dhaka, Bangladesh; 2 Centre for Health Economics Research and Evaluation (CHERE), University of Technology, Sydney (UTS), Sydney, Australia; 3 Department of Finance, Rawls College of Business, Texas Tech University, Lubbock, USA; 4 Department of Sociology, University of Alabama at Birmingham, Birmingham, USA; 5 Department of Public Health, Aarhus University, Aarhus, Denmark

**Keywords:** Antenatal care, Bangladesh, Concentration index, Concentration curve, Delivery care, Inequity, Maternal health

## Abstract

**Background:**

Universal access to health care services does not automatically guarantee equity in the health system. In the post Millennium Development Goals (MDGs) era, the progress towards universal access to maternal health care services in a developing country, like Bangladesh requires an evaluation in terms of equity lens. This study, therefore, analysed the trend in inequity and identified the equity gap in the utilization of antenatal care (ANC) and delivery care services in Bangladesh between 2004 and 2011.

**Methods:**

The data of this study came from the Bangladesh Demographic and Health Survey. We employed rate ratio, concentration curve and concentration index to examine the trend in inequity of ANC and delivery care services. We also used logistic regression models to analyse the relationship between socioeconomic factors and maternal health care services.

**Results:**

The concentration index for 4+ ANC visits dropped from 0.42 in 2004 to 0.31 in 2011 with a greater decline in urban area. There was almost no change in the concentration index for ANC services from medically trained providers during this period. We also found a decreasing trend in inequity in the utilization of both health facility delivery and skilled birth assistance but this trend was again more pronounced in urban area compared to rural area. The concentration index for C-section delivery decreased by about 33 % over 2004–2011 with a similar rate in both urban and rural areas. Women from the richest households were about 3 times more likely to have 4+ ANC visits, delivery at a health facility and skilled birth assistance compared to women from the poorest households. Women’s and their husbands’ education were significantly associated with greater use of maternal health care services. In addition, women’s exposure to mass media, their involvement in microcredit programs and autonomy in healthcare decision-making appeared as significant predictors of using some of these health care services.

**Conclusions:**

Bangladesh faces not only a persistent pro-rich inequity but also a significant rural-urban equity gap in the uptake of maternal health care services. An equity perspective in policy interventions is much needed to ensure safe motherhood and childbirth in Bangladesh.

## Background

Despite maternal health being a central element of the Millennium Development Goals (MDGs), the progress on improving maternal health indicators has been slower than expected in many developing countries. It has been estimated that in 2013 over a quarter million women died worldwide due to complications during pregnancy and childbirth [1]. The majority of these maternal deaths occurred in low and low-middle income countries because of unavailable, inaccessible, unaffordable or poor quality maternal health care services. Although the global maternal mortality rate (MMR) dropped by almost 50 % between 1990 and 2013, this is still far from the MDG 5 target of reducing maternal mortality by 75 % between 1990 and 2015. The average annual decline in global MMR between 1990 and 2013 was only 2.6 % against 5.5 % required to achieve MDG 5. Recent estimates show that only 11 countries are ‘on track’ to achieve the MDG 5 target and 63 countries are categorized as ‘making progress’ on this issue [1]. Although the average progress is satisfactory, there is a growing debate whether this progress has been inclusive and equitable.

Antenatal care (ANC) and delivery care services are the key components of safe motherhood. ANC services help pregnant woman and her family to interact with the formal health care system [2]. It also assists with the detection and treatment of pregnancy related complications and facilitates use of delivery and postnatal care services [3–5]. Quality delivery care by skilled health providers in hygienic environment and timely access to emergency care reduces the risk of death or serious complications for both the mother and the new born. ANC and delivery care have been found to have a strong positive associations with health outcomes for both mothers and children [6–8]. Process indicators like ANC and delivery care services provide easy, less costly and comparable information for monitoring progress in maternal health care [9, 10]. Inequity in maternal health may amongst other issues, reflect inequitable utilization of ANC and delivery care services. Therefore, identifying and addressing inequitable uptake of these services is important for improving maternal and child health.

Inequity in the use of maternal health services both between and within countries is a common phenomenon in developing countries [6, 11, 12]. Within the South Asian region, there is up to a 33 percentage-point gap between urban and rural areas in the coverage of births attended by skilled health personnel [13]. In addition, maternal deaths due to inadequate health care utilization are more pronounced among the less-educated, poor and rural population [14]. In the context of persistent inequity, policy attention has increasingly been devoted to equity within the development agenda [15, 16]. Furthermore, health system performance has been linked to its ability to ensure equitable access to essential services among different population groups [17, 18]. In accordance with this, the promotion of an “equity-oriented progressive realization of universal health coverage (UHC)” has been encouraged and measuring and monitoring equity in health care utilization is of utmost important [19]. Finally, geographic based analysis is important for monitoring equity in access to maternal health care services and it can help to design policy interventions for those areas in greatest need [19, 20]. As we are approaching the post-MDG era, it is essential to evaluate the progress towards achieving the goal of universal access to maternal health care services from an equity perspective.

In Bangladesh, the reduction of maternal mortality and expansion of safe motherhood services have been the central policy focus since the late 1990s. This is reflected in the Health and Population Sector Strategy (HPSS) and the rights’ based National Policy for Maternal Health of 2001. This policy placed greater importance on improving maternal health and advancing access to maternal health care in rural area. A central program feature has been to deliver an Essential Services Package (ESP) that includes comprehensive services for pregnancy, delivery and neonatal care at primary level health care facilities throughout the country [21]. The maternal health related MDG targets for Bangladesh include reducing MMR from 574 per 100,000 live births in 1990 to 143 per 100,000 live births by 2015 (a 75% reduction), increasing the proportion of births attended by skilled health personnel from 5% in 1990 to 50% by 2015 and obtaining universal coverage of at least one ANC visit by 2015. Over the last two decades, Bangladesh has made commendable progress in achieving these goals. For instance, MMR has dropped from 574 per 100,000 live births in 1990 to 170 per 100,000 live births in 2013 [22]. In addition, approximately 79% of pregnant women had at least one ANC visit to any healthcare provider in 2014 [23].

In spite of noticeable progress in the uptake of maternal health care services, inequity has become a central issue in Bangladesh in the last decade [20, 24, 25]. A common feature of the inequity in Bangladesh is that the use of maternal health care services has persistently been lower amongst the poorer and less educated, as well as those living in rural and remote areas [6, 26–28]. As Bangladesh moves forward to the post-MDG era, the pervasive inequity in the utilization of maternal health services should be systematically identified and addressed. Previous studies on maternal health service utilization in Bangladesh focused on socioeconomic, demographic and regional barriers to service utilization. These studies [29, 30] were limited by the small number of maternal healthcare indicators used, a focus on specific non-representative subpopulations [26, 31] and a lack of both absolute and relative equity assessments. Moreover, only a few studies have examined the trends in utilization of maternal health care services through an equity perspective.

The main objective of this study was to estimate the trend in inequity of utilization of maternal health care services over 2004–2011 in Bangladesh. Using a rich set of covariates, we also examined the role of different socioeconomic factors in ANC and delivery care services utilization. We contributed to the current literature on inequity of maternal health care services in Bangladesh in several ways. First, we provided updated estimates of trends in inequity of ANC and delivery care services using a comprehensive set of indicators. Second, we used both absolute and relative measures of inequity as well as regression analysis in a single study to capture different aspects of inequity in maternal healthcare service utilization. Finally, we presented a trend analysis of inequity in maternal health care utilization disaggregated at urban-rural level.

## Methods

### Data and analytic sample

This study used data from the three most recent rounds (2004, 2007 and 2011) of the Bangladesh Demographic and Health Survey (BDHS). This survey is a part of the long-standing worldwide Demographic and Health Survey (DHS) programme, which has become an important source of individual and household-level socio-demographic, health status and health care data in Bangladesh. BDHS is a nationally representative population based household survey conducted almost every three years in Bangladesh. BDHS collects information in the areas of maternal and child health, mortality, fertility, family planning and nutrition. Following multi-stage stratified sampling procedure, the survey collects data using a core set of survey questionnaires coherent with the MEASURE DHS model questionnaires, which ensure standardization and comparability of surveys across countries [32]. The survey maintains thorough data quality control procedures. Response rates in the three BDHSs were above 98 % and completeness of data was high for the variables of relevance to this study with missing information for less than 0.5 % cases (BDHS 2004, 2007 and 2011).

BDHS of 2004 and 2007 interviewed ever-married women aged 10–49 years while BDHS of 2011 interviewed ever-married women aged 12–49 years. We first limited the sample to women of reproductive ages (15–49 years). We then restricted the analysis to women who had at least one live birth in the three years preceding the survey. This allowed us to ensure consistency between outcome variables and explanatory variables and to minimize the time effects. If women had more than one live birth in the past three years, only care received for the most recent live birth was considered. Our final analytic sample consisted of 3730, 3365 and 4648 women aged 15–49 years in 2004, 2007 and 2011 respectively. We limited the analysis to the period 2004–2011 because our aim was to capture changes in the equity of the utilization of maternal health care after the policy change in 2001. Additionally, all indicators of maternal health care services and socioeconomic variables included in the analysis were only available in the latest three surveys.

### Outcome variables

The indicators of maternal health care services were divided into two categories: ANC and delivery care. ANC indicators include: a) 4+ ANC visits, b) receiving ANC from medically trained provider and c) receiving sufficient doses of tetanus toxoid (TT) injection during pregnancy. Delivery care indicators include: a) giving birth at a health facility, b) receiving skilled assistance at birth and c) birth delivered by caesarean section. Binary variables were created for each indicator as outlined below.

#### Antenatal care

Antenatal care services were assessed using three binary measures. The first ANC measure is whether women had at least four ANC visits. At least four ANC visits is the threshold level for adequate ANC coverage based on recommendations from the World Health Organisation (WHO) [33]. The second ANC measure is whether women received ANC from a medically trained provider. The third ANC measure is the receipt of adequate doses of tetanus (TT) injections, which is necessary to prevent maternal and neonatal deaths. A woman needs at least two doses of TT injection during pregnancy for full protection without prior dose of TT injection. Receipt of at least two doses of TT injection is a binary variable indicating whether a woman received 2+ TT injections during her last pregnancy.

#### Delivery care

Delivery care services were assessed using three binary measures. The first delivery care measure is whether women gave birth at a health facility (government or private hospital, government health centre, government health post, maternal and child welfare centre and NGO static clinic or sub-district health complex). The second delivery care measure is whether women received skilled birth assistance. This captures whether a skilled assistant or personnel attended a birth and this can be a doctor, a nurse/midwife, a family welfare visitor (FWV), or a community skilled birth attendant (CSBA). The third variable of delivery care services is whether a woman had a caesarean (C) section delivery. C-section is a measure of women’s access to skilled care for complicated and emergency deliveries.

### Predictor variables

The selection of explanatory variables in this study followed existing literature [26, 29, 30, 34] that documented significant association with different indicators of maternal health care. *Demographic characteristics* included woman’s age, woman’s age at first birth, parity, place of residence and region. We used women’s age as a continuous variable in this study while age at first birth is grouped into four categories. Typically, an overwhelming majority of women in Bangladesh gives birth to their first child before they reach their teen age (NIPORT et al., [35]). We, therefore, categorised age at first birth using 11–14 age as a reference group, to see whether differential age at first birth has any influence on maternal healthcare utilization. We also included parity measured by the reported number of living children.

*Socioeconomic characteristics* included woman’s education, woman’s employment, husband’s education and household wealth. Education is self-reported and BDHS collects the highest year of education completed by both women and their husbands. Employment is divided into two categories: currently unemployed and employed. Economic status is measured using the wealth index in the BDHS. The wealth index is a composite measure of a household’s cumulative living standard. The wealth index is calculated using easy-to-collect data on a household’s ownership of selected assets and dwelling characteristics, such as televisions and bicycles; materials used for housing construction; and types of water access and sanitation facilities [36]. This index places individual households on a continuous scale of relative wealth. Finally, all households are grouped into five wealth quintiles. Wealth quintiles are used to compare the influence of wealth on various population, health and nutrition indicators [37]. We also used women’s involvement in a micro credit programme as an indicator of a higher ability to pay for health care services [38, 39].

We also included *woman’s health care decision-making autonomy index* and *exposure to mass media index* following other studies [40, 41] from Bangladesh. To measure the aspects of household health care decision-making, the respondents were asked the following questions: 1) Who has the final say on the woman’s own health care? 2) Who has the final say on child health care? and 3) Can a woman go to the health centre alone or with her young children? The response options for the first two questions were: (a) respondent alone, (b) respondent and husband/partner, (c) respondent and other person, (d) husband/partner alone, (e) someone else and f) other. For the first two questions, a value of 1 was assigned if the response was (a), (b) or (c) and 0 for (d), (e) or (f). For the last question, a value of 1 was assigned if the response was ‘go to health centre alone’ or ‘go to health centre with young children’ and 0 was assigned if the response was ‘go to health centre with husband’ or ‘woman cannot go to health centre’. To create an index, we summed up all the scores for each respondent. The equally weighted summed score ranged from 0 to 3, with 0 indicating no participation and 3 indicating the highest level of participation. Finally, we created four categories of health care decision making autonomy with 0 representing ‘no’, 1 indicating ‘low’, 2 representing ‘medium’ and 3 representing ‘high’ autonomy. We constructed an index of exposure to mass media using women’s reporting of frequency of exposure to radio, television and newspapers in a typical week. For each medium, a value of 0 was assigned if the respondent did not have access to the medium at all, a value of 1 was assigned if the respondent used the medium less than once a week and a value of 2 assigned if the respondent used the medium at least once a week. We summed the scores for each medium and divided the score into three groups to create the mass media exposure index in which 0 indicates no exposure at all, 1–3 indicates irregular exposure and 4–6 indicates regular exposure.

### Statistical analysis

We estimated service coverage level in each survey year by tabulating our indicators of maternal health care service by wealth quintiles, place of residence and regions. We calculated richest (quintile 5) to poorest (quintile 1) and urban to rural ratios to present the absolute inequity in maternal health care service utilization. However, rate-ratios (richest/poorest and urban/rural) only take into account two extreme groups and only gauge absolute inequity. In other words, rate-ratios do not consider changes in the distribution of the population across socioeconomic groups. Therefore, we employed the concentration curve (CC) and the concentration index (CIX) as the standard equity measures to estimate wealth related inequity in use of each maternal health care service. We interpreted the inequity measured through the CC and the CIX as the horizontal inequity since every pregnant woman was assumed to be in equal need for maternal health care, e.g., skilled attendant at delivery, regardless of other background characteristics [42]. Horizontal equity in health care refers to “equal care for equal need irrespective of other characteristics such as income, race, religion, location, education, etc.”[43].

The concentration curve is a graphical way to present inequity in health care utilization and compare the level of inequity over time or across countries. The CC plots the cumulative proportion of individuals ranked by wealth quintiles (poorest first) against the cumulative proportion of a health care indicator. The line from the origin shows perfect equality. The further the CC away from the line of equality, the greater the degree of inequity. When we measured inequity in health care service utilization among women, the CC lying below the line of equality reflects disproportionate service utilization benefiting women from wealthy households. We plotted the CCs for each of the six indicators of maternal care from the BDHSs 2004, 2007 and 2011 in the same graph to compare the extent of inequity during 2004–2011. We also tested the statistical differences of the CCs of three surveys using multiple comparison approach (mca) and intersection union principal (iup) of dominance testing. The first method indicates statistical dominance even for one significant difference between curves in one direction while the second one concludes dominance only if there are significant differences at all points. A further explanation of dominance testing is described elsewhere [44].

Although the concentration curve is a useful tool for graphical presentation of inequity, it does not quantify the magnitude of inequity for convenient comparison over time. So, we used the concentration index, defined as twice the area between the CC and the line of equality, to measure the degree of inequity systematically associated with wealth [45]. The index takes a value between -1 and +1. A value of 0 indicates that the health care utilization is equally distributed across the socioeconomic groups [46]. A negative value of the CIX implies higher utilization among the poor (pro-poor) while a positive one indicates rich women have greater coverage than do poor women (pro-rich). In this paper, we used “convenient regression” formula proposed by [47] to calculate the concentration index. We also adjusted the sampling weight to compute the fractional rank variable to derive the standard error of the CIX of all six indicators of health care utilization variable. However, in case of a binary indicator, the value of the CIX may not lie between −1 and +1 as the lower and upper bounds of the CIX depend on the mean values of the variable [48]. Therefore, we normalized the CIX by dividing it by the reciprocal of the mean of outcome variable, since the mean of the outcome variable changes from one survey to the other survey [44].

We finally used logistic regression analysis to examine the effect of different correlates of utilization of maternal health care by pooling the three rounds of the BDHS data. We included survey fixed effect for each year to account for survey-specific differences. We checked the possibility of multi-collinearity among explanatory variables using variance inflation factors (VIFs) before entering the variables into pooled logistic regression analysis. Since the data in BDHSs were collected using a stratified multi-stage sampling method, we adjusted for individual sample survey weights and stratification in all estimations. Following the DHS Sampling and Household Listing Manual [49], we used the annual female population in Bangladesh from the Population Division of the United Nations [50] to calculate an appropriate weight for each observation in the analyses. We used STATA/SE V.12.0 (Stata Corp, College Station, Texas, USA) to conduct all statistical analyses in this study.

## Results

### Trend in maternal health service utilization

Table 1 reports an overview of the utilization of maternal health care services in Bangladesh during 2004–2011. The proportion of pregnant women who had 4+ ANC visits increased from about 17 % in 2004 to 26 % in 2011. The fraction of pregnant women who received ANC from medically trained personnel remained stagnant around 50 % during this period. The percentage of women taking 2 + TT during their pregnancy decreased from 60 % to 42 % during this same period. Delivery at a health facility increased from 12 % in 2004 to more than 29 % in 2011. We also found similar trend in skilled birth assistance and C-section delivery over the same period.

**Table 1:** Estimates of utilization of ANC and delivery care services in Bangladesh, 2004–2011

National	Antenatal Care									Delivery Care								
	(4+) ANC Visits (%)			Medically Trained ANC (%)			(2+) Tetanus Injection (%)			Health Facility Delivery (%)			Skilled Birth Assistance (%)			C-Section Delivery (%)		
	2004	2007	2011	2004	2007	2011	2004	2007	2011	2004	2007	2011	2004	2007	2011	2004	2007	2011
	17.13	22.32	26.03	49.24	51.46	52.69	60.03	55.23	42.21	12.12	18.60	29.21	12.63	18.72	26.32	5.74	9.61	17.23
Place of residence																		
Rural	11.73	17.58	19.82	43.52	45.45	45.74	59.72	55.42	40.52	7.71	13.10	23.02	7.54	13.35	20.80	2.34	6.75	13.77
Urban	36.70	38.32	44.80	72.92	69.96	70.91	63.22	52.08	46.82	28.14	33.94	49.48	27.43	33.75	44.57	14.15	18.40	28.87
Ratio: Urban to Rural	3.13	2.18	2.26	1.68	1.54	1.55	1.06	0.94	1.16	3.65	2.59	2.15	3.64	2.53	2.14	6.04	2.73	2.10
Region																		
Barisal	11.62	17.52	26.97	39.58	42.60	47.17	68.37	67.66	55.99	7.12	11.22	21.86	9.21	10.66	19.69	3.32	4.44	13.11
Chittagong	14.80	20.24	20.34	46.70	50.57	54.08	60.13	50.10	40.99	7.91	16.06	25.43	7.95	16.43	23.02	2.88	7.75	14.02
Dhaka	19.26	21.43	26.57	50.01	48.93	50.98	60.43	58.95	44.76	15.45	20.10	30.22	14.99	20.94	27.70	7.35	12.48	20.45
Khulna	19.23	27.89	33.34	56.95	65.18	61.29	54.62	50.82	34.02	18.87	27.11	45.70	18.16	27.87	39.91	5.44	11.85	26.29
Rajshahi	17.47	26.77	28.93	51.95	50.52	48.56	63.98	54.34	43.11	9.66	15.74	28.68	8.13	15.00	25.91	3.17	7.68	15.08
Sylhet	10.26	13.29	15.78	43.85	48.37	46.05	53.47	46.26	29.24	7.73	11.21	21.56	9.64	10.47	19.02	3.58	5.90	12.41
Wealth Index																		
Poorest	4.24	9.48	10.59	25.45	29.91	28.41	54.18	54.86	40.73	2.52	6.66	10.07	2.83	6.00	8.79	0.16	2.91	2.72
Poorer	7.07	11.37	14.90	39.14	36.26	36.78	56.87	55.35	44.88	3.34	5.63	16.61	3.26	5.21	13.63	0.79	1.61	9.25
Middle	13.01	15.45	20.95	49.44	44.78	49.89	60.78	54.83	42.27	6.76	9.99	24.71	7.55	11.16	22.21	2.16	4.29	14.52
Richer	19.54	27.97	32.83	62.99	63.28	63.50	65.94	53.56	36.54	14.84	20.71	39.90	13.40	20.93	35.46	4.73	9.69	23.03
Richest	47.99	49.36	53.31	82.46	83.84	85.59	67.61	54.83	45.86	38.31	48.58	60.02	36.88	48.99	56.54	19.07	30.01	41.07
Ratio: Richest to Poorest	11.33	5.21	5.03	3.24	2.80	3.01	1.25	1.00	1.13	15.21	7.29	5.96	13.04	8.17	6.43	119.13	10.31	15.10

### Inequity in maternal health service utilization

Table 1 also presents the changes in the utilization of ANC and delivery care between the richest and the poorest as well as urban and rural residents. Overall, the equity gap progressively narrowed between 2004 and 2011, mostly in C-section delivery, followed by health facility delivery, 4+ ANC visits and skilled birth assistance. The richest to poorest ratio in use of C-section delivery declined considerably from 119:1 in 2004 to 15:1 in 2011. This ratio also declined noticeably for health facility delivery from 15:1 in 2004 to 6:1 in 2011. However, the richest-poorest gap remained around 3:1 for receiving ANC services from a medically trained provider during 2004–2011. Utilization of maternal health care services was generally higher in urban area but the urban-rural ratio declined over time. The most equitable urban-rural coverage was for 2+ TT injection and ANC from a medically trained provider (Table 1). The urban-rural ratio sharply declined for C-section delivery, followed by skilled assistance at birth and health facility delivery. For example, the urban-rural ratio for delivery at health facility and skilled birth assistance declined to about 2.2 in 2011 from 3.6 in 2004. There were also regional variations in maternal health care service utilization. In terms of geographic region, the overall trend suggests that Sylhet division consistently lagged behind in uptake of maternal health care service compared to other divisions.

**Fig. 1:**
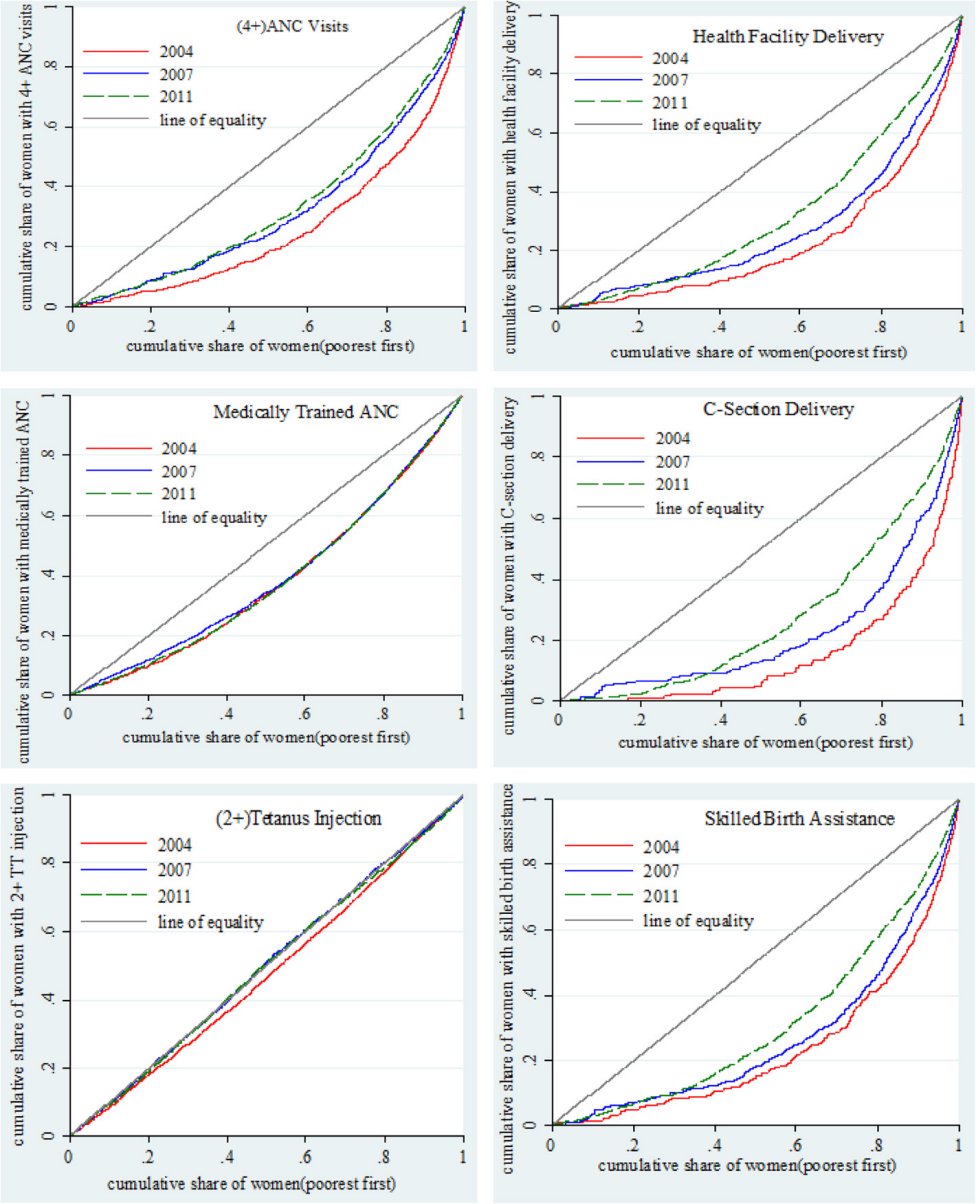
The concentration curves (CC) of ANC and delivery care services utilization in Bangladesh, 2004–2011. *Note:* A CC below line of equality shows pro-rich inequity. The further the CC away from the 45-degree line, the greater the degree of inequity

We presented the concentration curves for each indicator in Fig. 1 to provide a comparative picture of the trend in inequity of maternal health care utilization in Bangladesh during 2004–2011. The CCs are everywhere below the line of equality in all three surveys for every indicators, except for 2+ TT injections during pregnancy. In general, Fig. 1 demonstrates a disproportionate service utilization of important maternal health care services (other than 2+ TT injections) in Bangladesh between 2004 and 2011. However, there was a declining trend in inequity of maternal health service utilization except for ANC from a medically trained provider during the study period. This is depicted as the CCs for the rest of the indicators shifted closer to the line of equality over time. Table 2 presents the results of dominance testing of the statistical differences between the CCs for successive survey years as well as between the CCs from the earliest and most recent surveys. Findings from both approaches of dominance testing suggest that the CCs for every indicator (except ANC from medically trained provider) in 2011 exhibited a strong dominance over the CCs in 2004. This result indicates that inequity in utilization of maternal health care for most indicators decreased during 2004–2011.

**Table 2:** Test of dominance between concentration curves of ANC and delivery care utilization in Bangladesh, 2004–2011

(4+)ANC Visits	Year 1	Year 2	Significant Level	Rule	Result
	2004	2007	5 %	mca	2007 dominates 2004
	2004	2007	5 %	iup	Non-dominance
	2007	2011	5 %	mca	Non-dominance
	2007	2011	5 %	iup	Non-dominance
	2004	2011	5 %	mca	2011 dominates 2004
	2004	2011	5 %	iup	2011 dominates 2004
Medically Trained ANC	2004	2007	5 %	mca	Non-dominance
	2004	2007	5 %	iup	Non-dominance
	2007	2011	5 %	mca	Non-dominance
	2007	2011	5 %	iup	Non-dominance
	2004	2011	5 %	mca	Non-dominance
	2004	2011	5 %	iup	Non-dominance
(2+)Tetanus Injection	2004	2007	5 %	mca	2007 dominates 2004
	2004	2007	5 %	iup	Non-dominance
	2007	2011	5 %	mca	Non-dominance
	2007	2011	5 %	iup	Non-dominance
	2004	2011	5 %	mca	2011 dominates 2004
	2004	2011	5 %	iup	2011 dominates 2004
Health Facility Delivery	2004	2007	5 %	mca	Non-dominance
	2004	2007	5 %	iup	Non-dominance
	2007	2011	5 %	mca	2011 dominates 2007
	2007	2011	5 %	iup	Non-dominance
	2004	2011	5 %	mca	2011 dominates 2004
	2004	2011	5 %	iup	2011 dominates 2004
Skilled Birth Assistance	2004	2007	5 %	mca	Non-dominance
	2004	2007	5 %	iup	Non-dominance
	2007	2011	5 %	mca	2011 dominates 2007
	2007	2011	5 %	iup	Non-dominance
	2004	2011	5 %	mca	2011 dominates 2004
	2004	2011	5 %	iup	2011 dominates 2004
C-Section Delivery	2004	2007	5 %	mca	2007 dominates 2004
	2004	2007	5 %	iup	Non-dominance
	2007	2011	5 %	mca	2011 dominates 2007
	2007	2011	5 %	iup	Curves cross
	2004	2011	5 %	mca	2011 dominates 2004
	2004	2011	5 %	iup	2011 dominates 2004

*Note:* Dominance indicates CC in 1 year is significantly different from CC in other year while non-dominance implies insignificant difference between two CCs

We reported the concentration indices (CIX) with 95 % confidence interval (CI) at national level as well as for rural and urban areas in Table 3. The positive values of the CIX indicate evidence of the inequity in utilization of maternal care services in all three waves. At national level, the CIX for 4+ ANC visits dropped from 0.42 in 2004 to 0.31 in 2011 while it was around 0.20 for ANC from a medically trained provider in all years (Table 3). In 2004, the highest inequity was in C-section delivery utilization (CIX: 0.60) while there was almost no inequity in 2+ TT injections (CIX: 0.05). In 2011, CIXs were 0.40 and 0.01 for C-section delivery and 2+ TT injections respectively. Inequity in skilled birth assistance continued to decrease between 2004 and 2011. The CIXs for health facility delivery and C-section delivery decreased by about 33 % in 2011 compared to those in 2004. In general, we found evidence of inequity in utilization of maternal health services in Bangladesh but the extent of inequity lessened in recent years.

**Table 3:** The concentration index (CIX) for ANC and delivery care service utilization in Bangladesh, 2004–2011

		National			Rural			Urban		
	Year	CIX	(95 % CI)		CIX	(95 % CI)		CIX	(95 % CI)	
(4+)ANC Visits	2004	0.42	(0.41,	0.43)	0.38	(0.36,	0.39)	0.36	(0.34,	0.38)
	2007	0.32	(0.31,	0.34)	0.28	(0.27,	0.31)	0.32	(0.30,	0.33)
	2011	0.31	(0.30,	0.32)	0.27	(0.26,	0.28)	0.21	(0.20,	0.23)
Medically Trained ANC	2004	0.21	(0.21,	0.22)	0.21	(0.20,	0.21)	0.13	(0.13,	0.14)
	2007	0.20	(0.20,	0.21)	0.20	(0.19,	0.21)	0.14	(0.13,	0.15)
	2011	0.21	(0.21,	0.22)	0.22	(0.21,	0.22)	0.13	(0.12,	0.14)
(2+)Tetanus Injection	2004	0.05	(0.04,	0.05)	0.04	(0.04,	0.05)	0.06	(0.05,	0.07)
	2007	0.00	(−0.01,	0.01)	0.00	(−0.01,	0.01)	0.03	(0.02,	0.04)
	2011	0.01	(0.00,	0.01)	−0.03	(−0.04,	−0.02)	0.05	(0.04,	0.06)
Health Facility Delivery	2004	0.49	(0.47,	0.50)	0.45	(0.42,	0.47)	0.44	(0.41,	0.46)
	2007	0.41	(0.39,	0.43)	0.37	(0.34,	0.42)	0.38	(0.36,	0.40)
	2011	0.33	(0.32,	0.34)	0.32	(0.31,	0.33)	0.22	(0.21,	0.23)
Skilled Birth Assistance	2004	0.47	(0.45,	0.49)	0.41	(0.39,	0.43)	0.44	(0.42,	0.46)
	2007	0.42	(0.41,	0.43)	0.39	(0.36,	0.42)	0.40	(0.38,	0.42)
	2011	0.34	(0.34,	0.35)	0.33	(0.32,	0.34)	0.27	(0.25,	0.28)
C-Section Delivery	2004	0.60	(0.58,	0.63)	0.57	(0.54,	0.61)	0.47	(0.47,	0.47)
	2007	0.48	(0.46,	0.51)	0.47	(0.43,	0.51)	0.49	(0.47,	0.54)
	2011	0.40	(0.39,	0.41)	0.42	(0.41,	0.43)	0.35	(0.32,	0.37)

*Note:* A positive value of CIX indicates pro-rich horizontal inequity in maternal health care utilization. The higher the value of CIX, the greater is inequity

We also found a similar picture from the disaggregated analysis at rural and urban level. The CIX for health facility delivery were 0.45 in rural area and 0.44 in urban area in 2004 while it became 0.32 and 0.22 for respective areas in 2011. Findings from Table 3 clearly indicate that urban area outperformed rural area in reducing inequity in maternal health care service utilization over the period of 2004–2011. In other words, there was an overall progress in the reduction of inequity in these indicators of maternal health care services but rural area always lagged behind to achieve equity compared to urban area.

### Multivariate analysis

Before moving to the multivariate analysis, we presented descriptive statistics of the demographic, socio-economic and other predictor variables for each survey round in Table 4. Means of categorical variables in this table indicates proportions. There was almost no change in average age of women and the distribution of residents across area (almost 70 % in rural area) and division of residence, income quintile and microcredit involvement. We found that the proportion of women giving first birth at very early age (11–14 years) decreased from 0.14 in 2004 to 0.09 in 2011. Findings from Table 4 also indicate that the percentage of women with 3 or more children went down (45 % in 2004 to 34 % in 2011) over the 7 years. The percentage of women with no decision-making autonomy reduced from 25 % in 2004 to 14 % in 2011, while the percentage of women with at least some say on own and children’s health and being able to go to the health clinic (at least with someone else) have increased from 22 % in 2004 to 36 % in 2011. Although average number of years of education for both women and husband increased between 2004 and 2011, women’s participation in formal employment declined to 8 % from 16 % in this period (Table 4).

**Table 4:** Summary statistics of predictor variables used in logistic regression analysis

	2004		2007		2011	
Variable	Mean	Std. Dev.	Mean	Std. Dev.	Mean	Std. Dev.
Current Age (in years)	24.75	6.22	24.73	5.94	24.49	5.64
Age at first birth (11–14)	0.13	0.33	0.09	0.28	0.09	0.28
Age at first birth (15–19)	0.67	0.47	0.65	0.48	0.64	0.48
Age at first birth (20–24)	0.17	0.38	0.22	0.42	0.22	0.42
Age at first birth (25–42)	0.03	0.18	0.04	0.20	0.05	0.21
Parity (1)	0.30	0.46	0.34	0.47	0.37	0.48
Parity (2)	0.25	0.43	0.27	0.44	0.29	0.46
Parity (3+)	0.45	0.50	0.39	0.49	0.34	0.47
Area of Residence						
Rural	0.70	0.46	0.64	0.48	0.68	0.47
Urban	0.30	0.46	0.36	0.48	0.32	0.47
Division of Residence						
Barisal	0.11	0.31	0.12	0.33	0.11	0.32
Chittagong	0.22	0.41	0.21	0.40	0.20	0.40
Dhaka	0.22	0.41	0.22	0.41	0.16	0.37
Khulna	0.13	0.34	0.12	0.32	0.12	0.32
Rajshahi	0.19	0.39	0.17	0.37	0.25	0.43
Sylhet	0.13	0.34	0.17	0.37	0.15	0.36
Wealth quintile						
Poorest	0.23	0.42	0.19	0.39	0.21	0.41
Poorer	0.19	0.39	0.20	0.40	0.19	0.39
Middle	0.19	0.39	0.18	0.39	0.19	0.39
Richer	0.18	0.38	0.19	0.39	0.20	0.40
Richest	0.22	0.41	0.23	0.42	0.20	0.40
Microcredit involvement						
No	0.71	0.45	0.66	0.48	0.68	0.47
Yes	0.29	0.45	0.34	0.48	0.32	0.47
Mass media exposure						
No	0.31	0.46	0.37	0.48	0.35	0.48
Irregular	0.10	0.31	0.07	0.26	0.14	0.35
Regular	0.59	0.49	0.56	0.50	0.51	0.50
Health care decision making autonomy						
No autonomy	0.25	0.43	0.12	0.33	0.14	0.35
Low	0.24	0.43	0.23	0.42	0.20	0.40
Medium	0.28	0.45	0.33	0.47	0.30	0.46
High	0.22	0.42	0.31	0.46	0.36	0.48
Employment						
No	0.84	0.36	0.78	0.41	0.92	0.28
Yes	0.16	0.36	0.22	0.41	0.08	0.28
Own education (in years)	4.00	3.84	5.24	4.32	5.80	3.81
Husband’s education (in years)	4.38	4.49	5.06	4.85	5.50	4.62

Table 5 presents the odds ratios (OR) of utilization of ANC and delivery care indicators with 95 % confidence interval (CI) from the logistic regression analysis. We found no statistical association between ANC indicators and *women’s current age* but the relationship between *women’s current age* and each indicator of delivery care was highly significant. However, the likelihood of greater utilization of delivery care at higher age is small. Women with higher *age at first birth* were more likely to have delivery at health facility, skilled assistance at birth and delivery by C-section than women who gave their first birth at younger age (Table 5). For instance, women who gave their first birth at 25 years or more were about 3 times more likely to have delivery at health facility and delivery by C-section than women who gave first birth at 14 years or less. Results from Table 5 also indicate that women with higher *parity* consistently used less maternal care services than those with parity one. For example, women with 3 or more living children were about three times less likely than women with one living child to have 2+ TT injections, delivery at health facility, skilled assistance at birth and delivery by C-section. Women with three or more living children were about 40 % less likely to have 4+ ANC visits and ANC from a medically trained provider than women with one living child. The association is very strong for both ANC and delivery care services.

**Table 5:** Multivariate logistic regression of ANC and delivery care utilization in Bangladesh, 2004–2011

Antenatal Care				Delivery Care		
Variable	(4+)ANC Visits	Medically Trained ANC	(2+)Tetanus Injection	Health Facility Delivery	Skilled Birth Assistance	C-Section Delivery
	OR (95 % CI)	OR (95 % CI)	OR (95 % CI)	OR (95 % CI)	OR (95 % CI)	OR (95 % CI)
Age (in years)	1.00 (0.99,1.02)	1.02^*^ (1.00,1.03)	1.01 (1.00,1.02)	1.04^***^(1.02,1.06)	1.04^***^(1.02,1.07)	1.05^***^ (1.02,1.08)
Age at first birth (Ref = 11–14)						
Age at first birth (15–19)	0.77^*^(0.61,0.97)	1.02 (0.88,1.18)	0.84^*^ (0.72,0.97)	1.16 (0.91,1.48)	1.10 (0.86,1.42)	1.48^*^ (1.00,2.19)
Age at first birth (20–24)	0.91 (0.69,1.22)	1.09 (0.90,1.32)	0.76^**^ (0.63,0.92)	1.45^*^ (1.08,1.94)	1.44^*^ (1.07,1.93)	2.14^***^ (1.39,3.28)
Age at first birth (25–42)	1.12 (0.72,1.74)	1.04 (0.75,1.44)	0.90 (0.65,1.24)	2.74^***^ (1.78,4.24)	2.41^***^ (1.58,3.69)	3.37^***^ (1.93,5.90)
Parity (Ref = 1)						
Parity (2)	0.83^*^(0.72,0.97)	0.70^***^(0.61,0.80)	0.51^***^ (0.45,0.58)	0.59^***^ (0.50,0.69)	0.57^***^(0.48,0.67)	0.64^***^ (0.53,0.78)
Parity (3+)	0.59^***^(0.47,0.75)	0.57^***^(0.47,0.68)	0.31^***^ (0.25,0.37)	0.36^***^ (0.28,0.48)	0.38^***^(0.29,0.51)	0.37^***^ (0.25,0.54)
Urban (ref = rural)	1.80^***^ (1.53,2.12)	1.56^***^ (1.35,1.81)	0.96 (0.85,1.08)	1.69^***^ (1.43,1.99)	1.54^***^ (1.31,1.81)	1.12 (0.92,1.35)
Division (ref = Barisal)						
Chittagong	0.78 (0.59,1.04)	1.19 (0.97,1.46)	0.57^***^ (0.47,0.70)	1.13 (0.87,1.46)	1.08 (0.85,1.38)	1.07 (0.79,1.44)
Dhaka	0.96 (0.74,1.24)	1.15 (0.94,1.39)	0.65^***^ (0.53,0.79)	1.61^***^ (1.25,2.07)	1.57^***^ (1.24,1.99)	1.96^***^ (1.44,2.67)
Khulna	1.13 (0.86,1.49)	1.59^***^ (1.27,1.99)	0.43^***^ (0.35,0.53)	2.61^***^ (2.02,3.38)	2.36^***^ (1.86,2.99)	1.90^***^ (1.39,2.61)
Rajshahi	1.56^**^ (1.19,2.03)	1.48^***^ (1.20,1.82)	0.60^***^ (0.49,0.74)	1.78^***^(1.38,2.29)	1.56^***^ (1.23,1.98)	1.52^**^ (1.12,2.06)
Sylhet	0.74 (0.55,1.00)	1.39^*^ (1.07,1.82)	0.43^***^ (0.34,0.54)	1.20 (0.91,1.58)	1.15 (0.89,1.48)	1.24 (0.89,1.71)
Wealth quintile (Ref = Poorest)						
Poorer	1.00 (0.78,1.30)	1.15 (0.99,1.34)	1.04 (0.90,1.20)	0.92 (0.70,1.19)	0.87 (0.65,1.15)	1.41 (0.91,2.19)
Middle	1.24 (0.98,1.56)	1.44^***^ (1.22,1.70)	1.05 (0.91,1.22)	1.18 (0.91,1.52)	1.30 (1.00,1.70)	1.92^**^ (1.26,2.92)
Richer	1.70^***^ (1.33,2.19)	1.98^***^ (1.65,2.36)	1.00 (0.84,1.18)	1.82^***^ (1.41,2.34)	1.82^***^ (1.41,2.36)	2.63^***^ (1.72,4.03)
Richest	2.91^***^ (2.24,3.78)	4.06^***^ (3.25,5.06)	1.26^*^ (1.04,1.52)	3.16^***^ (2.40,4.17)	3.32^***^ (2.51,4.38)	4.89^***^ (3.17,7.54)
Microcredit involvement (Ref = No)	1.22^**^ (1.07,1.38)	1.14^*^ (1.02,1.27)	1.01 (0.91,1.12)	1.10 (0.95,1.26)	1.08 (0.93,1.25)	1.10 (0.91,1.32)
Mass media exposure (Ref = No)						
Irregular	1.04 (0.83,1.29)	1.10 (0.94,1.28)	1.09 (0.94,1.27)	1.09 (0.86,1.37)	1.10 (0.87,1.39)	1.29 (0.94,1.76)
Regular	1.27^*^ (1.06,1.51)	1.27^***^ (1.11,1.44)	1.06 (0.95,1.18)	1.26^*^ (1.04,1.52)	1.21^*^ (1.01,1.45)	1.36^*^ (1.06,1.73)
Health care decision making autonomy (Ref = No)						
Low	1.21^*^ (1.00,1.46)	1.33^***^ (1.15,1.55)	1.01 (0.88,1.16)	1.04 (0.85,1.27)	1.09 (0.89,1.34)	1.28 (0.99,1.68)
Medium	1.36^**^ (1.13,1.64)	1.29^***^ (1.12,1.48)	1.03 (0.91,1.17)	1.00 (0.82,1.21)	1.06 (0.87,1.29)	1.28 (0.99,1.64)
High	1.53^***^ (1.28,1.84)	1.51^***^ (1.30,1.75)	1.11 (0.96,1.28)	1.05 (0.87,1.28)	1.12 (0.92,1.36)	1.10 (0.86,1.41)
Employment (Ref = No)	0.86 (0.72,1.03)	1.03 (0.88,1.19)	0.94 (0.83,1.08)	0.77^*^ (0.63,0.95)	0.82^*^ (0.67,0.99)	0.74^*^ (0.58,0.96)
Own education ((in years))	1.12^***^ (1.09,1.14)	1.11^***^ (1.09,1.13)	0.96^***^ (0.94,0.98)	1.12^***^ (1.09,1.15)	1.11^***^ (1.08,1.14)	1.10^***^ (1.07,1.13)
Husband’s education (in years)	1.05^***^ (1.04,1.07)	1.05^***^ (1.04,1.07)	1.00 (0.99,1.02)	1.06^***^ (1.04,1.08)	1.06^***^ (1.04,1.08)	1.08^***^ (1.05,1.10)
Survey fixed effect (Ref = 2004)						
Survey 2 (Year = 2007)	1.10 (0.90,1.33)	0.79^**^ (0.67,0.93)	0.77^***^ (0.67,0.88)	1.35^**^ (1.10,1.64)	1.40^***^ (1.15,1.70)	1.74^***^ (1.39,2.17)
Survey 3 (Year = 2011)	1.29^**^ (1.09,1.52)	0.76^***^ (0.66,0.89)	0.44^***^ (0.38,0.50)	2.91^***^ (2.41,3.51)	2.47^***^ (2.05,2.97)	4.03^***^ (3.27,4.95)

*Notes:* 1) Significant at * *p* < 0.1; ** *p* < 0.05; *** *p* < 0.01, 2) Odds ratio was adjusted for all other explanatory variables

We found that *urban* women had higher probability of receiving maternal health services than their rural counterparts. For example, women in urban area were 50 % or more likely than women living in rural area to receive ANC from medically trained provider and skilled assistance at birth. Urban women were about 80 and 70 % more likely than rural women to have 4+ ANC visits and delivery at health facility, respectively. Again, these relationships were significant at 1 % level. There were persistent *divisional* differences in the utilization of maternal health services. For example, uptake of C-section delivery was almost twice as high in Dhaka (OR = 1.96) and Khulna division (OR = 1.90) compared to Barisal division. Skilled birth assistance was 57 % higher in Dhaka (OR = 1.57) and 56 % higher in Rajshahi (OR = 1.56) compared to Barisal. These findings were highly significant. Women from Khulna, Rajshahi and Sylhet division had significant and higher probability of getting medically trained ANC compared to Barisal.

Regression results in Table 5 indicate that Women from the *wealthier* households were consistently more likely to utilize maternal care services than those from the poorer households. For example, women from the richest households (quintile 5) were about 3 times more likely than women from the poorest households (quintile 1) to have 4+ ANC visits (OR = 2.91, 95 % CI: 2.24–3.78), delivery at health facility (OR = 3.16, 95 % CI: 2.40–4.17) and skilled birth assistance service (OR = 3.32, 95 % CI: 2.51–4.38). Women from the richest households had four times higher probability than women from the poorest households to receive ANC from a medically trained provider. Furthermore, women from the richest households are about five times (OR = 4.89, 95 % CI: 3.17–7.54) more likely than women from the poorest households to have C-section delivery. We found that statistical significance level became stronger with higher wealth quintiles. Our findings specify that the women’s and their husbands’ *education* were significantly associated with greater use of maternal health care services. The association between delivery care indicators and women’s *employment* was not very strong. Additionally, there was no significant relationship between indicators of ANC and women’s employment.

Women’s *exposure to mass media* appears to be significantly associated with maternal care service utilization. Odds ratios in Table 5 reveal that women with regular exposure to mass media exposure were 27 % more likely than women with no media exposure to have 4+ ANC visits, ANC from a medically-trained provider (significant at 1 % level) and delivery at health facility. Women with higher media exposure were 36 % (significant at 10 % level) more likely than women with no media exposure to have births delivered by C-section. Women’s *autonomy in healthcare decision-making* was significantly associated with increased utilization of ANC services. For example, women with higher autonomy were 50 % more likely to have both ANC from a medically trained provider and 4+ ANC visits than women with no autonomy. *Micro credit involvement* also had positive association with ANC services but we found no significant relation with delivery care indicators. Survey specific odds ratios indicate that use of several maternal health services noticeably increased over the last seven years. Most improvement is found in using C-section delivery (significant at 1 % level), followed by skilled birth assistance and health facility delivery. For example, odds of giving birth by C-section increased by 4 times in 2011 compared to 2004. Rather slow progress is observed in the utilization of recommended 4+ ANC in 2011 (OR = 1.29).

## Discussion

Bangladesh has achieved marked improvement in different indicators of maternal health during the last decade [51]. However, there exists not only a large gap between rich and poor women but also significant urban-rural differences in access to and use of maternal health care services in Bangladesh [51, 52]. Hence, this study analysed the trend in inequity of the utilization of maternal health care services using the three recent rounds of the Bangladesh Demographic and Health Survey.

The general finding is that coverage level of maternal health care service increased for all outcomes except for 2+ TT injections during 2004–2011. Since the country has almost achieved universal coverage (9 out of 10 women) for TT injections before pregnancy in 2011, it is not unusual to find a reduction in usage of 2+ TT injections during pregnancy [23]. Between 2004 and 2011, the coverage level of delivery care at health facilities and skilled birth assistance more than doubled while 4+ ANC visits and receiving ANC from a medically trained provider increased only by 9 and 3 % respectively. Study finding indicates that the rate of increase in selected indicators of maternal health care service utilization was always lower in rural area than in urban area. However, this gap in improvement had been slowly narrowing over time. When we disaggregated our analysis at regional level, we found that Sylhet division always performed worst while Khulna division led all divisions in almost all indicators. It is evident that the proportion of women using ANC services increased during 2004–2011 in Bangladesh but this progress is slow and is highly unlikely to reach the target of 50 % coverage for 4+ ANC visits by 2016, a goal set by Health Population Nutrition Sector Development Programme in Bangladesh. Only one fourth of pregnant women had the WHO recommended 4+ ANC visits in 2011. Findings from this study also reveal that there are significant regional differences in the coverage of 4+ ANC visits. The general trend is that Khulna division topped the list and Sylhet division remained as worst performer in uptake of 4+ ANC visits and ANC from a medically trained provider. It was perhaps due to some hard-to-reach and remote hill-tract areas in Sylhet and Chittagong divisions, where service coverage is consistently low. In addition, religious conservatism and low level of literacy often characterize many communities in these low-performing regions.

Despite low coverage and inequity in adequate ANC visits and ANC from a medically trained provider, progress of TT vaccination among women during pregnancy was excellent in Bangladesh. There had been almost equitable distribution in case of 2+ TT injection among women in their last pregnancy since 2004. This finding is similar to the results of the study by Karim et al. 2006 [53] and they argued that the narrowing gap between poor and non-poor in this indicator is due to the availability of this service free of charge and easy accessibility. Analysis of the concentration curves and the concentration indices reveals a pro-rich and a pro-urban inequity in uptake of the rest five indicators of maternal health care. We also found that inequity in delivery care services was higher than inequity in ANC services. Our analysis highlights that low utilization of maternal health care is more evident among women with lower socioeconomic status and is consistent with results from similar studies [6, 29, 54, 55]. These findings imply that richer and urban households were more able and willing to pay for services from the increasingly widespread private health facilities while poorer and rural households were consistently disadvantaged in affording and accessing the needed care.

In our study, women with better education and educated husbands were more likely to utilize ANC services; perhaps because educated women are more likely to realize the benefits of using maternal healthcare services [56]. Studies also documented that education increases female autonomy and decision-making power within the household and consequently influences the uptake of maternal health care services [56, 57]. Our findings document that women exposed to mass media were more likely to use maternal health care services. It is possibly due to the increased dissemination of health education messages through popular mass media. We found that microcredit membership of the woman and women’s decision-making autonomy in family health affairs are significantly associated with the increased use of ANC, which is also in line with previous Bangladeshi studies [26, 39, 40]. The experience from Bangladesh shows that poor mothers are likely to contribute to household resources through their microcredit participation and thus increase the households’ ability to pay for health services [26]. Microcredit institutions also play complementary roles in providing their members with health service related information, which increase women’s access to and timely utilization of needed services. With respect to demographic factors*nn* parity appears to affect utilization negatively. Previous studies also reported similar association in many developing countries [58–60]. One possible explanation for the low utilization among high parity women is that women with higher number of births usually tend to develop confidence due to their experience and knowledge accumulated from previous pregnancies and births and therefore, they are less motivated to opt for services from health facilities or health professionals in developing countries [61].

Earlier studies identified a number of potential barriers to access to professional delivery care in Bangladesh. The most significant of these barriers include costs of services, long distance to a health facility, long-standing tradition of using local untrained traditional birth attendants and various cultural barriers such as stigma, purity, women’s limited mobility especially due to Muslim institution of *parda* and preference of women to give birth at home [62]. Community based home-care service approach in which obstetric care provided by trained community health workers have been found to be effective in reducing neonatal mortality and improving maternal and neonatal care practices [63]. Targeted interventions that create enabling environment by expanding the services and providing incentive schemes have been very effective in benefiting the poorer segments of society in utilizing critical maternal care services in many communities in Bangladesh, Cambodia, Kenya and Mexico [64–67]. Besides, mobilizing services through community health workers have been reported to be effective in improving equity in maternal care services in Bangladesh [68]. We suggest policy makers to continue to scale up such effective incentive schemes and community based programme efforts targeting poorer section of population in Bangladesh.

We finally take a note about the questionable progress in the proportion of births delivered by C-section. The high prevalence of C-section delivery at national level (17 % of all deliveries in 2011) exceeding WHO recommended threshold level (5–15 %) indicates that many of these surgeries are likely to be clinically unnecessary [69]. The downside of high rate of C-section delivery in poor countries such as Bangladesh is that these countries have limited capacity to provide safe surgical births. As a result, if unnecessary C-section continues, many women who actually need this service will be pushed out of the health care system [70]. Unnecessary C-section can therefore have undesirable implications for health equity in developing countries. Future research may consider exploring the causes of high prevalence of such expensive services in the poor countries. Policy makers should particularly focus on designing monitoring programmes and surveillance mechanisms that could identify at what points of health care system and in what circumstances unnecessary C-sections are used.

The main strength of this study is that we analysed six indicators of maternal health care services to examine the overall progress through an equity lens. However, our study is not beyond limitations. First, our analysis is limited to examine the trend over the seven-year, as data on all six indicators were not available in the earlier rounds of BDHS. Second, we cannot draw any causal interpretation from regression analysis due to cross sectional nature of the data. Finally, we have not extended our analysis to decompose the contributions of different socioeconomic factors in inequity because we covered broad aspects of maternal health care utilization over time. Further studies may also consider exploring the possible reasons of regional variation in inequity.

## Conclusion

Average country progress may not result in sustainable health outcome if the improvement remains inequitable. Assessing the trend in inequity of maternal health services in a low-income country like Bangladesh is important for policy design to accelerate the overall progress as well as to narrow the equity gap in access to and use of essential maternal health care services. Our findings suggest a persistent increase in uptake of ANC and delivery care services in Bangladesh between 2004 and 2011. Nevertheless, maternal health care utilization rates were quite low, which makes it difficult to relate to the large decrease in maternal mortality in Bangladesh in the last decade. As far as the MDG target on reduction of maternal mortality is concerned, a recent countdown study showed that Bangladesh is likely to achieve it [70]. However, equitable progress of different indicators of maternal health care services studied in this paper is not satisfactory. This study highlights that the country faces not only a persistent pro-rich in equity but also a significant rural-urban inequity in the uptake of maternal health care services. In addition, certain population groups with low socioeconomic status are consistently disadvantaged in utilizing the needed services. These findings have important implications for strengthening the health care delivery system. Maternal health care system in Bangladesh requires developing culture-sensitive and need-based delivery approaches especially for underserved poor women, women from hard-to-reach, remote hill-track and rural area. It is high time to design multi-sectoral and concerted interventions from an equity perspective to ensure safe motherhood and childbirth in Bangladesh.

## Abbreviations

ANC, Antenatal care; CC, Concentration curve; CI, Confidence interval; CIX, Concentration index; C-section, caesarean section; DHS, Demographic health survey; ESP, Essential Services Package; MDG, Millennium Development Goal; MMR, maternal mortality rate;

OR, Odds ratio; TT, Tetanus toxoid; UHC, Universal health coverage; WHO, World Health Organisation.
